# Tofacitinib treatment alters mucosal immunity and gut microbiota during experimental arthritis

**DOI:** 10.1002/ctm2.163

**Published:** 2020-09-23

**Authors:** Julie Hablot, Maroua Ferhat, Aonghus Lavelle, Fatouma Salem, Mahdia Taieb, Jasna Medvedovic, Nadège Kindt, Pascal Reboul, Frédéric Cailotto, Jean‐Yves Jouzeau, Gérard Eberl, Harry Sokol, David Moulin

**Affiliations:** ^1^ CNRS, IMoPA Université de Lorraine Nancy France; ^2^ Sorbonne Université, INSERM Centre de Recherche Saint‐Antoine, CRSA Paris France; ^3^ Gastroenterology Department, Saint Antoine Hospital Assitance Publique‐Hopitaux de Paris (APHP) Paris France; ^4^ INRA UMR1319 Micalis & AgroParisTech, Jouy en Josas France; ^5^ Paris Center for Microbiome Medicine (PaCeMM) Paris France; ^6^ Microenvironment & Immunity Unit Institut Pasteur Paris France; ^7^ INSERM U1224 Paris France; ^8^ Département de Pharmacologie Clinique et Toxicologie Centre Hospitalier Régional Universitaire de Nancy Vandoeuvre‐les‐Nancy France; ^9^ Centre Hospitalier Régional Universitaire de Nancy Contrat d'interface Vandoeuvre‐les‐Nancy France

Dear Editor,

Tofacitinib is a potent and orally active inhibitor of the JAK family (with high selectivity to JAK‐1 and JAK‐3), which suppresses inflammatory signaling of γc‐chain cytokines that have been implicated in the pathogenesis of autoimmune diseases and represents an interesting therapeutic option in rheumatoid arthritis (RA). Similar to other biologic disease‐modifying antirheumatic drugs (DMARDs), treatment with tofacitinib is associated with increased infections frequency. Thus, monitoring of absolute leukocyte counts (ALC) is recommended to assess infection risk in tofacitinib‐treated RA patients.^1^

Here, we report the impact of tofacitinib treatment on mucosal immunity and gut microbiota diversity during collagen‐induced arthritis in DBA1/J mouse, a Gold‐standard model of experimental RA.

Mucosal immunity acts as a double agent: a real firewall against pathogens, but also a partner or an ally that coevolved to monitor, and control the gut microbiota. Moreover, commensal microorganisms impact the development of the immune system itself and help in the maintenance of critical physiological processes including host defense.^2^ There are many recent arguments supporting the role of the gut microbiota in the pathogenesis of rheumatic diseases (reviewed in Ref. 3). Experiments in germ‐free or gnotobiotic conditions have provided a deeper understanding of host‐microbial interactions and demonstrated that gut bacteria can induce autoimmunity in genetically predisposed animal models.^4^ In humans, dysbiosis may contribute to RA and spondyloarthritis (SpA) pathogenesis by altering mucosal integrity, which could facilitate the migration of bacteria, or their metabolites into the joints, or by impairing the ability of the mucosal immune system to induce protective immunity.^5^ Indeed, intestinal dysbiosis has been reported in patients with early or established RA.

As shown in Figure 1A‐C, curative treatment with tofacitinib reduced drastically the severity of arthritis without affecting disease incidence. This anti‐arthritic effect is associated with a decrease in Th17 lymphocytes, which are central players in the clearance of extracellular infections and fungi but also in the pathogenesis of RA and other auto‐immune disease. Accordingly, Th17 cell number in draining popliteal and inguinal lymph nodes was increased in arthritic conditions (data not shown) but also in *lamina propria* from the small intestine (siLP) and in mesenteric lymph nodes (mLN) (Figure 1D). Tofacitinib treatment abolished arthritis‐induced siLP and mLN Th17 cell overabundance. Of note, ILC3s, the innate lymphoid counterpart of Th17, were also increased in gut from arthritic mice compared to healthy controls. Tofacitinib treatment blocked arthritis‐induced ILC3 increase in siLP and mLN (Figure 1E). These results echo a recent publication from Marco Colonna's group demonstrating that a spontaneous mutation in the exon 14 of the JAK3 gene (translating into a noncatalytic mutant) conferred to mice a pan‐ILC deficiency.^6^ Interestingly, Robinette et al observed that tofacitinib was able to inhibit ILCs proliferation and differentiation, ascribing this effect to their IL‐7 and IL‐15 dependence for development (IL‐7 and IL‐15 signaling pathways are mediated through JAK3 activation).^6^

**FIGURE 1:**
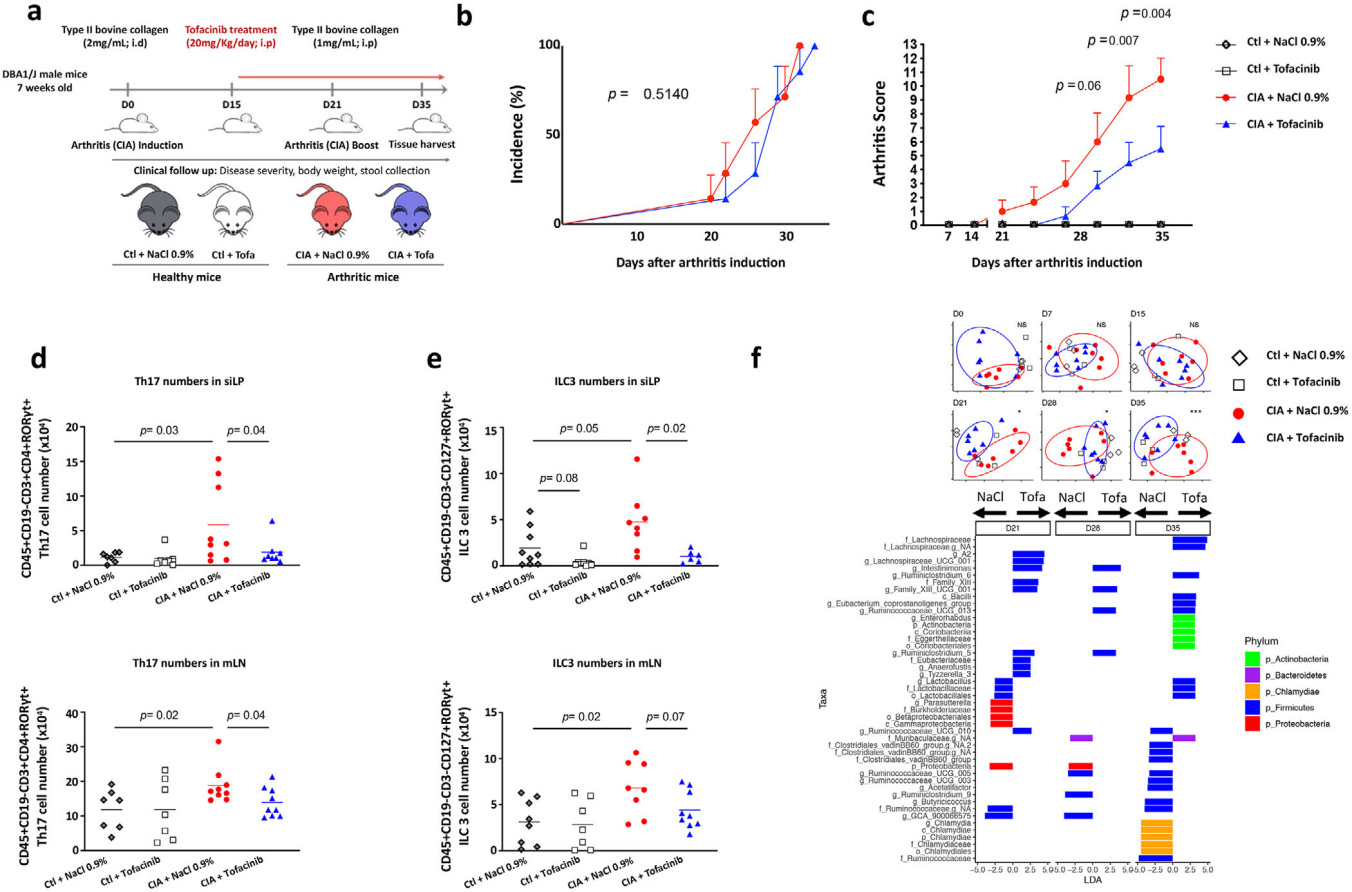
**A**, Heterologous collagen‐induced arthritis (CIA) was induced in 7 weeks old DBA/1J male mice by an intradermal injection (i.d) of type II bovine collagen (2 mg/mL) emulsified in complete Freund's adjuvant at the base of the tail. At day 21, a booster immunization was performed by an intraperitoneal (i.p) injection of type II collagen (1 mg/mL) in incomplete Freund's adjuvant. Tofacitinib was administered daily (20 mg/kg/day) by i.p injection from arthritis onset (from day 15) until necropsy (day 35). Mice were divided into four groups: (1) vehicle‐treated control; (2) tofactinib‐treated control; (3) vehicle‐treated arthritic, and (4) tofactinib‐treated arthritic mice. Data are compiled from eight mice in each group from three independent experiments. **B and C,** Disease incidence (B) and disease severity (C) were monitored and scored twice a week from arthritis onset until necropsy. Data are expressed as mean ± SEM for each group. Statistical differences between groups were evaluated using log‐rank test for incidence and using Mann‐Whitney *U* test for arthritis score. **D**, Th17 cell number in small intestine *Lamina propria* (siLP, upper panel) and mesenteric lymph nodes (mLN, lower panel) assessed by flow cytometry after tofacitinib treatment in arthritic mice. Data are expressed as mean ± SD for each group. Statistical differences between groups were evaluated using an ANOVA and Fisher's PLSD post hoc test. **E,** ILC3 cell number in small intestine *Lamina propria* (siLP, upper panel) and mesenteric lymph nodes (mLN, lower panel) assessed by flow cytometry after tofacitinib treatment in arthritic mice. Data are expressed as mean ± SD for each group. Statistical differences between groups were evaluated using an ANOVA and Fisher's PLSD post hoc test. **F**, Tofacitinib effects on microbiota. Upper panel, PCoA plots of Bray‐Curtis divergence for the six times. Significance stars refer to PERMANOVA comparing clustering of arthritic mice treated with placebo (NaCl) or treated with tofacitinib. Control mice are included in the plots for reference. Lower panel: barplots of significantly different taxa by Lefse, comparing arthritic mice given placebo (NaCl) and those given tofacitinib at the three later time points (D21, D28, and D35)

Although ILC3s are important players of mucosal homeostasis, notably through IL‐22 production, their chronic activation is deleterious. Thus, during *Helicobacter hepaticus* infection and anti‐CD40‐mediated colitis, ILC3s drive inflammation in an IL‐23‐dependent manner. In Crohn's disease, patients exhibit an increased frequency of NCR^−^ CCR6^+^ ILC3s. Whether ILC3s contribute to RA, explaining some of the anti‐arthritic activity of Tofacitinib, remains to be explored.

We subsequently analyzed the gut microbiota by 16S sequencing and demonstrated that although tofacitinib did not affect alpha‐diversity, alterations in beta‐diversity were apparent from day 21, in the periods of greatest disease activity (Figure 1F, PCoA plots of Bray‐Curtis distance). Interestingly, when these time points were compared by linear discriminant analysis with effect size (LEfSe),^7^ we noted a decrease in potentially pathogenic members of the phyla Proteobacteria and Chlamydiae in tofacitinib‐treated mice, as well as an increase in some beneficial members of the Firmicutes and Actinobacteria phyla (Figure 1F).

To conclude, we provide here preclinical data showing that tofacitinib affects both mucosal immunity and gut microbiota. Our study raises several questions. Are some anti‐arthritic effects of tofacitinib ILC3s or microbiota driven? Are rare infections observed upon Jak inhibitors a consequence of mucosal immunity disruption in a dysbiotic context?

Taken together, these preclinical data also pave the way for microbiota study in tofacitinib‐treated patients aiming at identifying theragnostic fecal biomarkers.

## CONFLICT OF INTEREST

The authors declared no conflict of interest.

## AUTHOR CONTRIBUTIONS

All authors were involved in drafting the article or revising it critically for important intellectual content, and all authors approved the final version to be published.

## Supporting information

Supporting information

Supporting information
