# Supplementary material for: Tofacitinib treatment alters mucosal immunity and gut microbiota during experimental arthritis
Source: Clin Transl Med. 2020 Sep 23;10(5):e163. doi: 10.1002/ctm2.163 (PMC7510778; doi:10.1002/ctm2.163)
Supplement: Supplementary file 1 — Supporting information [file CTM2-10-e163-s001.pdf]

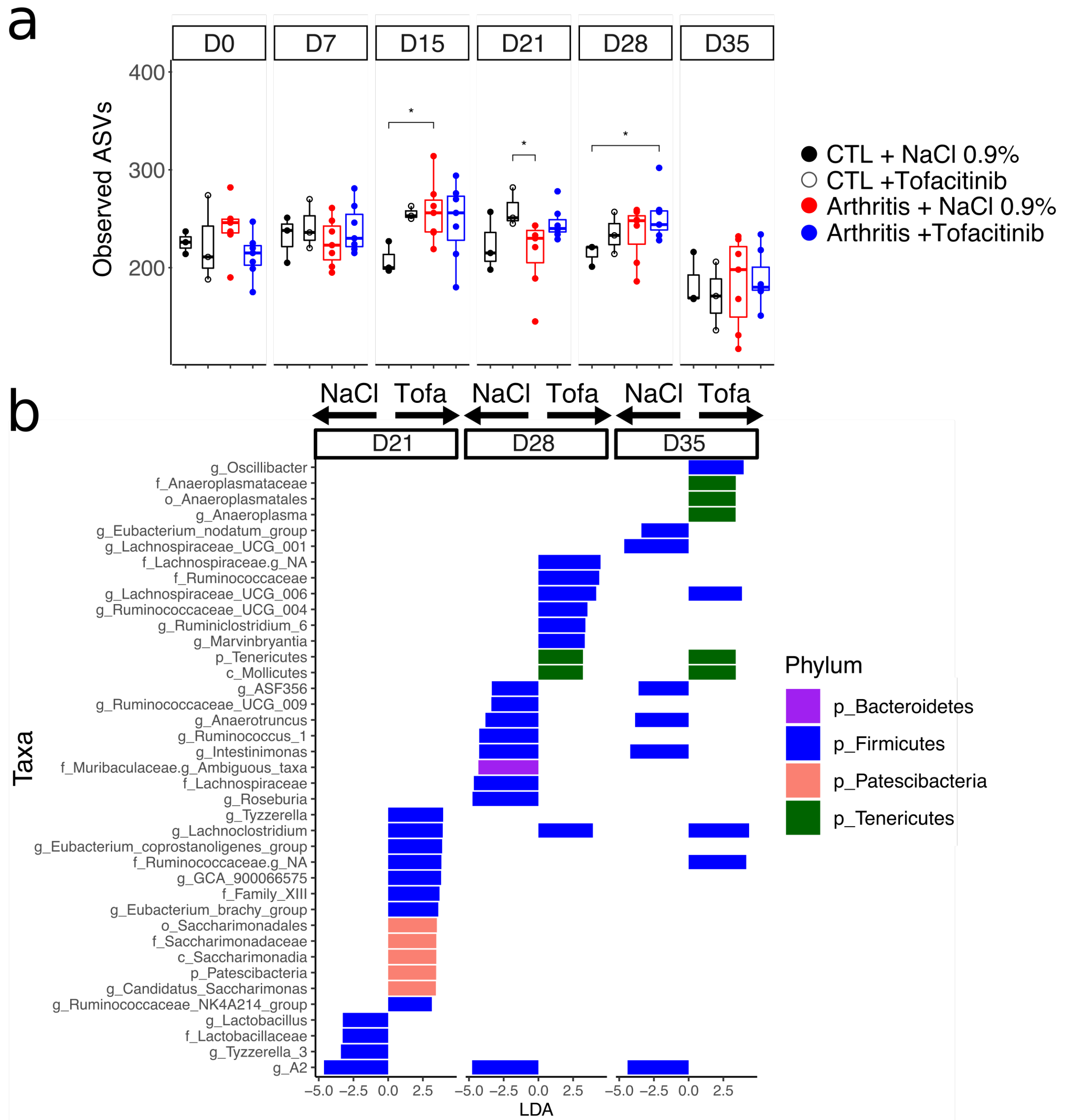

Supplementary Figure 1. a. There was no consistent differences in alpha diversity between groups and across time points. Here the number of observed ASVs are plotted (amplicon sequence variants). b. Barplot of taxa significantly different between control groups given NaCl and tofacitinib at the same time points presented in Figure 1f.
