# Supplementary material for: Tofacitinib treatment alters mucosal immunity and gut microbiota during experimental arthritis
Source: Clin Transl Med. 2020 Sep 23;10(5):e163. doi: 10.1002/ctm2.163 (PMC7510778; doi:10.1002/ctm2.163)
Supplement: Supplementary file 2 — Supporting information [file CTM2-10-e163-s002.pdf]

Supplementary figure legend

a. There was no consistent difference in alpha diversity between groups and across time points. Here the number of observed ASVs (amplicon sequence variants) are plotted.
b. Bar plots of taxa significantly different between control groups given NaCl and tofacitinib at the same time points presented in Figure 1f.

Materials and methods

Induction of collagen arthritis and treatment regimen

The experimental protocol has been approved by the Animal care and Use regional Committee (CELMEA, No 14762) and strictly adhere to national and European recommendations. Collagen-induced arthritis (CIA) was induced in 7-weeks old DBA/1 male mice. At day 0, arthritis was induced by an intradermal injection of 100µL of 2mg/ml type II bovine collagen (Mdbiosciences, Zurich, Switzerland) emulsified in complete Freund's adjuvant (CFA) at the base of the tail. At day 21, a booster intra-peritoneal injection of 100µL of 1mg/ml type II collagen in incomplete Freund's adjuvant has been performed.
Compounds were prepared in the vehicle (0.9% Saline) and sonicated to create a very fine aqueous suspension. Tofacitinib was administered daily at a dose of 20 mg/kg/d.
Arthritic controls and naïve controls received vehicle only. Dosing of all mice has been initiated on day 1 before onset of arthritis (from day 15) and continued until day 35. Non-arthritic groups (vehicle-treated and Tofacitinib treated) are used to monitor Tofacitinib effects alone.
Four experimental groups of 8 mice (vehicle-treated control, Tofactinib treated control, vehicle-treated arthritic, Tofactinib treated arthritic mice) have been analyzed in each experiment. Animals were housed in solid-bottomed plastic cages with access to tap water and standard rodent pelleted chow (Scientific Animal Food & Engineering A04) *ad libitum*. Cage effect was controlled by cohousing animals receiving the same treatment (4 animals per cage, 2 cages per experimental group for each of the 3 experiments).
In each experiment, 3 mice were used for FACS analysis.

Clinical assessment of arthritis

Arthritis severity was evaluated three times per week on each paw with a score ranging from 0 to 4, for a maximal score of 16 by animal (0 = normal joint, 1 = moderate redness and slight swelling or swelling of one or two digits, 2 = moderate redness and moderate paw swelling or swelling more than three digits, 3 = redness and swelling from tarsal joints to metatarsal joints, 4 = severe redness and severe swelling of the entire paw).

Isolation of cells from Small Intestinal *lamina propria* and Flow cytometry analysis

Small intestines were excised and transferred to ice-cold PBS. Adipose tissue and Peyer's patches were surgically removed. For flow cytometry analysis experiments, tissues were opened and the lumen contents removed by shaking in cold PBS. Epithelial cells were

removed by shaking tissue (200 rpm) in EDTA buffer (5mM EDTA) for 20 min at 37°C. Pieces of tissue were washed with PBS and digested in Liberase TL (25mg/mL; Roche), DNaseI (50mg/mL, Sigma Aldrich) and 2% FCS DMEM (GIBCO) solution at 37°C for 30 min with a shaking rotator (80 *g*). Remaining tissue was mechanically dissociated and the lymphocytes were separated by Percoll gradient. Mesenteric Lymph Node (MLN) were surgically removed and grinded on a 70µM filter mesh. Cells from small intestine and MLN were counted on an automated cell counter before cell staining.

FACS analysis

For FACS analysis, cells were stained with antibodies to the following markers: -CD4, -RORγt, -Tbet, -CD19, -CD3, -CD45, -GATA3, -CD25 and antibodies to differentiate each cell population. All antibodies were purchased from BD, eBioscience or Biolegend. Dead cells were excluded from analysis using AquaZombie dye (eBioscience). For experiment involving intracellular staining, cells were fixed and permeabilized using Foxp3 staining kit (ebioscience). Gallios cytometer (Beckman) was used for cell acquisition and the flow cytometry data were analyzed with Kaluza software. Cells were stained with combination of antibodies specific of ILC3 and Th17 subset.

Microbiota analysis

Stool collection and DNA extraction

Faecal samples were homogenized and aliquots of 0.2g were frozen at −80°C for subsequent analysis. DNA was extracted from faecal samples using a previously described method[1]. In brief, following both mechanical and chemical microbial lysis, nucleic acid precipitation was performed in isopropanol for 10 min at room temperature, with 15 minutes of incubation on ice, followed by centrifugation for 30 min at 20 000 g and 4°C. The resulting pellets were suspended in 450 µL of phosphate buffer and 50 µL of potassium acetate. Following RNase treatment and DNA precipitation, nucleic acids were recovered via centrifugation at 20 000 g and 4°C for 30 min. The DNA pellet was then suspended in 80 mL of trypsin-EDTA buffer.

Sequencing

Amplicon sequencing of the V3-V4 region of the 16S ribosomal RNA gene was performed using the primers – 16S sense 5′-TACGGRAGGCAGCAG-3′ and anti-sense 5′-CTACCNGGGTATCTAAT-3′ – according to an optimized and standardized 16S amplicon library preparation protocol (Metabiote, GenoScreen, Lille, France). In brief, 16S PCR was performed using 5ng of genomic DNA according to the protocol at Metabiote and using bar-coded primers (Metabiote MiSeq Primers). This was used at a final concentration of 0.2 µmol/L, (annealing temperature of 50°C for 30 cycles). PCR product purification was performed using the Agencourt AMPure XP-PCR purification system (Beckman Coulter, Brea, CA, USA) and was subsequently quantified according to the manufacturer's protocol. Samples were multiplexed at equal concentrations. 250 bp paired-end sequencing was performed on an Illumina MiSeq platform (Illumina, San Diego, CA, USA) using the protocol at GenoScreen. Raw paired-end reads were subjected to: (1) quality filtering using the

PRINSEQ-lite PERL script[2], by truncating the bases from the 3′ end, that did not exhibit a quality <30, based on the Phred algorithm and (2) searching for and removing both forward and reverse primer sequences using CutAdapt, with no mismatches allowed in the primer sequences. Only sequences with perfectly matching forward and reverse primers were included.

16S sequence analysis

Amplicon sequence variants (ASVs) were determined in the Qiime2 environment (version 2020.2) [3] using the dada2 algorithm [4, 5]. The Silva reference database (version 132)[6] was used for taxonomic classification. Subsequently, data was imported into the R statistical environment (R version 3.6.1)[7]. ASVs that were present in only a single sample, as well as those that could not be assigned to a Phylum-level taxonomy, were filtered. Data was analyzed with the phyloseq package (version 1.28.0)[8]. Raw sequence data are accessible in the Sequence Read Archive (accession number pending).

Alpha diversity was assessed using the number of observed ASVs and pairwise comparisons were tested using the Wilcoxon rank sum test. Analysis of beta diversity was performed on proportion-normalized data using the Bray-Curtis divergence. Assessment for significant differences in beta diversity between arthritis mice treated with placebo and arthritis mice treated with tofacitinib were tested using PERMANOVA with the adonis function in the vegan package[9] (version 2.5-6) in R with 9999 permutations. Principle co-ordinate analysis (PCoA) plots included all samples, although the PERMANOVA significance tests refer just to differences between groups in mice with arthritis. Differential abundance was tested using linear discriminant analysis with effect size (Lefse), at the default settings[10]. The R packages ggplot2 (version 3.2.1)[11] and ggpubr (version 0.2.3)[12] were used for plotting microbiota analysis.

1. Sokol, H., et al., Fungal microbiota dysbiosis in IBD. Gut, 2017. 66(6): p. 1039-1048.
2. Schmieder, R. and R. Edwards, Quality control and preprocessing of metagenomic datasets. Bioinformatics, 2011. 27(6): p. 863-4.
3. Bolyen, E., et al., Reproducible, interactive, scalable and extensible microbiome data science using QIIME 2. Nature Biotechnology, 2019. 37(8): p. 852-857.
4. Callahan, B.J., et al., DADA2: High-resolution sample inference from Illumina amplicon data. Nature Methods, 2016. 13: p. 581.
5. Callahan, B.J., et al., Bioconductor Workflow for Microbiome Data Analysis: from raw reads to community analyses. F1000Res, 2016. 5: p. 1492.
6. Quast, C., et al., The SILVA ribosomal RNA gene database project: improved data processing and web-based tools. Nucleic Acids Research, 2012. 41(D1): p. D590-D596.
7. R, R: A language and environment for statistical computing. 2018, R Foundation for Statistical Computing, Vienna, Austria. URL http://www.R-project.org/.
8. McMurdie, P.J. and S. Holmes, phyloseq: An R Package for Reproducible Interactive Analysis and Graphics of Microbiome Census Data. PLOS ONE, 2013. 8(4): p. e61217.
9. Oksanen, J., et al., vegan: Community Ecology Package. R package version 2.4-5., 2017.

10. Segata, N., et al., Metagenomic biomarker discovery and explanation. Genome Biology, 2011. 12(6): p. R60.
11. Wickham, H., ggplot2: Elegant Graphics for Data Analysis. 2009: Springer Publishing Company, Incorporated. 216.
12. Kassambara, A., ggpubr: 'ggplot2' Based Publication Ready Plots. R package version 0.2.3. 2019.
